# Small-molecule TREM2 agonists: From artificial intelligence driven discovery to therapeutic application in Alzheimer’s disease

**DOI:** 10.4103/NRR.NRR-D-25-01478

**Published:** 2026-02-05

**Authors:** Sungwoo Cho, Moustafa Gabr

**Affiliations:** Department of Radiology, Molecular Imaging Innovations Institute (MI3), Weill Cornell Medicine, New York, NY, USA

The Alzheimer’s disease (AD) therapeutic landscape is evolving rapidly. While anti-amyloid antibodies have achieved regulatory approval, their incremental clinical benefits have intensified interest in neuroinflammation as a complementary therapeutic axis. Triggering receptor expressed on myeloid cells 2 (TREM2) represents a particularly attractive microglial target, given that loss-of-function variants confer a three-fold elevation in AD risk. Although TREM2 antibodies have shown promise in preclinical studies, clinical translation is hindered by fundamental limitations: restricted blood–brain barrier (BBB) penetration (< 0.1%), prohibitive annual costs exceeding $100,000, and pharmacokinetic inflexibility that precludes dose adjustment. Here, we examine how artificial intelligence (AI)-assisted approaches have enabled the discovery of small molecule TREM2 agonists and their implications for AD treatment.

**TREM2 in Alzheimer’s disease — genetic risk and mechanistic rationale for therapeutic targeting:** TREM2 orchestrates protective microglial responses through DAP12-mediated signaling, activating pathways that regulate metabolic reprogramming, survival, and phagocytosis (Deczkowska et al., 2020). Genetic evidence firmly establishes its therapeutic relevance: the R47H variant confers AD risk comparable to Apolipoprotein E ε4 heterozygosity, while loss-of-function impairs plaque compaction, reduces amyloid-beta clearance, and prevents adoption of disease-associated microglial states. Conversely, TREM2 activation enhances amyloid clearance and preserves cognitive function in animal models (Wang et al., 2015; Schlepckow et al., 2020).

The initial focus on antibody based TREM2 agonists yielded promising preclinical results, with enhanced plaque clearance and improved behavioral outcomes. However, the recent Phase 2 failure of AL002 despite achieving target engagement highlights fundamental limitations. Large molecular weight of antibodies restricts BBB penetration, limiting access to parenchymal microglia. Their extended half-lives (21–28 days) prevent dose adjustment based on disease stage, which is problematic given evidence that TREM2 activation timing critically influences outcomes (Lee et al., 2018). Manufacturing costs and requirements for intravenous administration further limit accessibility, particularly in resource-limited settings where most dementia patients reside.

**Comparative advantages of small molecules over antibodies and associated development challenges:** Small molecules offer compelling advantages such as efficient BBB penetration, oral bioavailability, flexible dosing, and dramatically lower production costs. However, structural complexity of TREM2 has hindered development. The receptor lacks obvious druggable pockets, with ligand binding occurring across a broad, shallow surface stabilized by disulfide bonds. Traditional screening yielded hit rates below 0.01%, with poor selectivity and weak potency.

The breakthrough came with VG-3927, demonstrating that small molecules could achieve functional TREM2 activation (Mirescu, 2024). This compound binds an allosteric site, triggering conformational changes that enhance signaling with nanomolar cellular potency. Subsequent optimization yielded compounds with improved pharmacokinetics, validating the small molecule approach while highlighting the need for novel discovery strategies.

**Figure 1:**
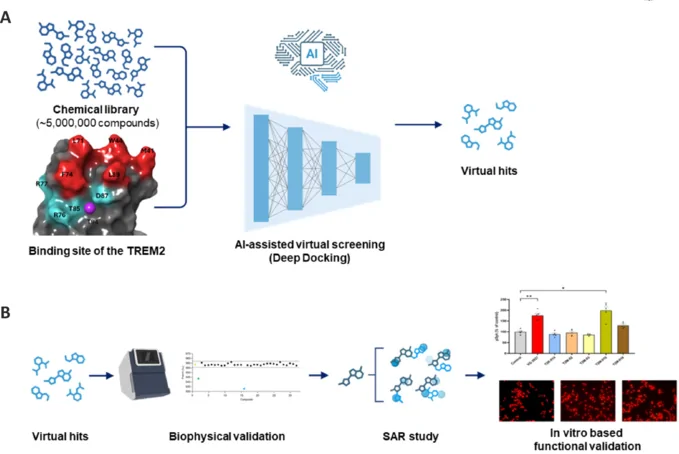
AI-assisted discovery pipeline for TREM2 small molecule agonists. (A) Deep Docking-based virtual screening of ~5 million compounds against the TREM2 binding site. (B) Hit validation through biophysical binding assays, SAR optimization, and functional characterization showing SYK phosphorylation (graph) and enhanced microglial phagocytosis (microscopy: IBA1 in red, beads in green), leading to identification of T2M-010 (Kd = 0.83 μM). Created with BioRender.com. Unpublished data from Cho et al., 2025. AI: Artificial intelligence; IBA1: ionized calcium-binding adapter molecule 1; SAR: structure-activity relationship; SYK: spleen tyrosine kinase; TREM2: triggering receptor expressed on myeloid cells 2.

**Application of artificial intelligence to overcome traditional drug discovery limitations:** To address these challenges, we implemented the Deep Docking platform (**Figure 1**), an AI-augmented virtual screening workflow integrating physics-based molecular docking with iteratively trained Quantitative Structure-Activity Relationship neural networks (Cho et al., 2025). This architecture reduces computational requirements approximately 50-fold, enabling practical screening of multimillion-compound libraries. To address conformational flexibility of TREM2, we employed ensemble docking against multiple receptor conformations from molecular dynamics simulations. Screening 5.2 million compounds yielded 30 high-confidence hits, with one confirmed binder (T2K-014; Kd = 28.88 μM) corresponding to a 3.3% experimental hit rate that substantially exceeds the < 0.01% typically achieved by conventional screening against shallow protein-protein interaction surfaces. While rigorous benchmarking against contemporary machine-learning baselines (graph neural networks, 3D-Convolutional Neural Network) and traditional docking protocols (Glide, AutoDock Vina) with standardized enrichment metrics (Area Under the Receiver Operating Characteristic Curve, Boltzmann-Enhanced Discrimination of Receiver Operating Characteristic) remains an important future direction, successful identification of a novel chemotype from this exceptionally challenging target supports AI-guided prioritization utility.

Experimental validation confirmed T2K-014 as a genuine TREM2 binder (Kd = 28.88 μM) with a structurally distinct indole-pyridine scaffold. Systematic structure-activity relationship studies exploring 27 analogs revealed critical requirements such as the indole moiety proved essential, while the pyridine region tolerated modifications. This optimization yielded T2M-010 with submicromolar affinity (Kd = 0.83 μM), representing a 35-fold improvement (Cho et al., 2025).

**Molecular mechanisms and functional consequences of small molecule-mediated TREM2 activation:** T2M-010 engages TREM2 through a mechanism distinct from antibodies. Molecular docking suggests binding within a hydrophobic pocket formed by W44, L71, and L89 residues, stabilizing an active receptor conformation. Notably, biophysical studies have demonstrated that multiple AD-relevant ligands engage overlapping surfaces on TREM2 (Greven et al., 2025), raising the possibility that T2M-010 may similarly occupy a functionally conserved binding region. However, this proposed allosteric binding mode remains a computational hypothesis requiring experimental validation. Orthogonal biophysical techniques (surface plasmon resonance, isothermal titration calorimetry) are needed to determine kinetic parameters (kon, koff) and binding stoichiometry. Site-directed mutagenesis of predicted contact residues (W44A, L71A, L89A) will establish their contribution to binding energy. Structural techniques, including HDX-MS or cryo-EM, are needed to confirm the binding epitope and allosteric mechanism.

Functionally, T2M-010 induces SYK phosphorylation comparable to benchmark agonist VG-3927 and enhances BV2 microglial phagocytosis by 167% (Cho et al., 2025). However, TREM2-dependent specificity requires validation in knockout or knockdown microglia, or with TREM2/DAP12-blocking antibodies. Parallel assessment of Toll-like receptor and Fc-gamma receptor pathways will exclude off-target immunostimulation and assay artifacts. Extension to human induced pluripotent stem cell-derived microglia and patient cells harboring risk variants (R47H) will strengthen translational confidence. Preclinical ADME (Absorption, Distribution, Metabolism, Excretion) profiling revealed favorable CNS characteristics: high BBB permeability (PAMPA-BBB Pe = 9.2 × 10^–6^ cm/s), negligible efflux liability (ratio = 1.07), moderate hepatic stability (t_1/2_ = 68.4 minutes), and acceptable cardiac safety margins (human ether-a-go-go-related gene IC_50_ > 60 μM). Nevertheless, *in vivo* studies establishing brain exposure, dose-proportionality, and correlation between plasma concentrations and soluble triggering receptor expressed on myeloid cells 2 (sTREM2) biomarkers remain critical next steps (Cho et al., 2025).

**Impact on neurovascular unit integrity and regional brain microglial heterogeneity:** Beyond microglial activation, TREM2 signaling influences neurovascular unit integrity. Evidence suggests TREM2 maintains BBB function through pericyte-endothelial interactions, with deficiency associated with increased vascular permeability (Sweeney et al., 2019). Uniform brain distribution of small molecules enables comprehensive targeting of both perivascular and parenchymal microglia, potentially addressing vascular contributions to cognitive decline affecting most AD patients.

The temporal dynamics of TREM2 activation prove critical for therapeutic efficacy. Early intervention enhances protective responses, while late-stage activation might exacerbate inflammation (Gratuze et al., 2018). Unlike antibodies with extended half-lives that preclude rapid dose adjustment, small molecules enable precise titration to match optimal therapeutic windows. This control becomes particularly important given regional variations in microglial activation, with hippocampal microglia showing earlier TREM2 upregulation than cortical populations.

**Strategic opportunities for combination therapies and personalized treatment approaches:** Small-molecule TREM2 agonists offer unique opportunities for combination therapy. Co-administration with anti-amyloid antibodies could enhance plaque clearance while mitigating antibody-related imaging abnormalities through improved microglial function (Salloway et al., 2022). The distinct mechanisms and non-overlapping toxicities support rational combination development. Similarly, pairing with BACE inhibitors could reduce amyloid production while enhancing clearance.

The oral bioavailability enables innovative trial designs including adaptive dosing based on biomarkers, treatment holidays to prevent tolerance, and rapid dose adjustment for adverse events. Given the temporal sensitivity of TREM2 activation, trials should employ preregistered adaptive designs and enrichment strategies targeting patients most likely to benefit. Statistical approaches, such as PROCOVA-MMRM (Prognostic Covariate Adjustment with Mixed Model for Repeated Measures), could increase precision and substantially reduce sample size requirements by incorporating validated prognostic scores including baseline sTREM2 levels, Apolipoprotein E genotype, and amyloid burden into longitudinal efficacy analyses. Prevention trials in at-risk populations, impractical with expensive antibodies, become feasible. The cost advantages of small molecule therapeutics carry substantial public health implications, given that the majority of the global dementia burden is concentrated in resource-constrained healthcare systems.

**Critical considerations for clinical translation, including biomarker development and safety monitoring:** A robust biomarker strategy should integrate longitudinal sTREM2 monitoring as a pharmacodynamic readout, next-generation microglial positron emission tomography tracers targeting colony stimulating factor 1 receptor or purinergic receptor P2Y12, and colony stimulating factor proteomics panels to capture microglial state transitions. First-in-human studies should establish pharmacokinetics/pharmacodynamics relationships using soluble TREM2 as the primary endpoint, with adaptive dosing based on biomarker responses. Safety monitoring requires slow-dose titration to minimize inflammatory responses, scheduled dose holidays to prevent receptor desensitization, and predefined stopping rules based on colony stimulating factor cytokine thresholds. Critically, oral small molecules afford temporal control achievable within hours versus weeks for antibodies, enabling rapid dose adjustment if adverse signals emerge.

**Future perspectives and conclusion:** The convergence of AI based drug discovery and neuroimmunology has enabled breakthrough advances in small-molecule TREM2 agonist development. T2M-010 demonstrates that computationally driven approaches can overcome structural challenges previously considered intractable, achieving functional receptor activation with favorable drug-like properties. Success in clinical translation would provide accessible, orally administered therapies modulating neuroinflammation, a fundamental advance beyond current antibody approaches.

These findings position small-molecule TREM2 agonists as more than scientific curiosities; they represent a viable path toward accessible neuroinflammation-targeting therapies. The advantages inherent to small molecules, including dosing flexibility and combination potential, enable treatment personalization impossible with current biologics. Successful clinical translation would fundamentally advance AD therapeutics, bringing disease-modifying intervention within reach of patients worldwide.

*This work was supported by the National Institutes on Aging under grant number R01AG083512 (to MG).*
